# Effect of ultrasound-guided serratus anterior plane block in pediatric patients undergoing pectus bar removal: a retrospective study of selected cases

**DOI:** 10.3389/fped.2025.1600442

**Published:** 2025-08-01

**Authors:** Lianzhe Wu, Zenghua Xu, Xuemei Zhang, Fang Wang

**Affiliations:** ^1^ Department of Ultrasound, Beijing Children’s Hospital, Capital Medical University, National Center for Children’s Health, Beijing, China; ^2^ Department of Anesthesiology, Beijing Children’s Hospital, Capital Medical University, National Center for Children’s Health, Beijing, China

**Keywords:** serratus anterior plane block, postoperative analgesia, pediatrics, pectus bar removal, retrospective study

## Abstract

**Objective:**

To evaluate the effect of ultrasound-guided bilateral serratus anterior plane block (SAPB) in selected pediatric patients undergoing pectus bar removal, focusing on those with anticipated higher pain sensitivity or risk of opioid-related complications.

**Methods:**

A retrospective analysis was conducted on pediatric patients aged 6–17 years who underwent elective pectus bar removal under general anesthesia between September 2024 and February 2025. Patients were categorized into two groups: those receiving ultrasound-guided bilateral SAPB (Group S, *n* = 30) based on clinical factors such as pain sensitivity or risk of opioid-related complications, and a control group without SAPB (Group C, *n* = 46). Pain scores at rest and during coughing were assessed using the Numerical Rating Scale (NRS) immediately after awakening (Aldrete score ≥ 9) and at 1, 3, 6, 12, and 24 hours postoperatively. General patient information, dosages of sufentanil, propofol, and remifentanil, postoperative fentanyl supplementation, surgical duration, anesthesia duration, extubation time, and adverse events such as postoperative nausea and vomiting were recorded.

**Results:**

Group S exhibited significantly lower incidences of postoperative hypoxemia and lower resting and coughing NRS scores within 6 hours postoperatively compared to Group C (*P* < 0.008, Bonferroni-corrected). SAPB significantly reduced perioperative opioid use (*P* < 0.05). No statistically significant differences were observed in postoperative nausea and vomiting, delayed ambulation, or delayed oral intake (*P* > 0.05).

**Conclusion:**

In selected pediatric patients undergoing pectus bar removal, SAPB, as part of a multimodal analgesia regimen, was associated with improved perioperative analgesia, reduced postoperative hypoxemia, and decreased opioid use. While SAPB provides clear benefits in reducing postoperative pain and opioid use, we do not suggest routine implementation in all patients. Instead, its use should be individualized based on patient-specific factors such as pain sensitivity, previous analgesic response, and risk of opioid-related complications.

## Introduction

The incidence of pectus excavatum has a ratio between 0.1 and 0.8 per 100 persons (1, 2). The minimally invasive Nuss procedure, which corrects sternal deformities by inserting a pectus bar into the thoracic cavity, is currently the most commonly used surgical technique for treating moderate to severe pectus excavatum (3). Pectus bar removal, a surgical procedure typically performed 2–3 years post-Nuss procedure, can cause varying degree of postoperative pain (4). While some patients experience mild discomfort, others may have significant pain due to factors such as pain sensitivity, surgical complexity, or individual response to analgesics, necessitating advanced pain management strategies. Intravenous analgesics alone may provide insufficient pain relief or lead to adverse effects such as respiratory depression, dizziness, nausea and vomiting. The serratus anterior plane block (SAPB) is an effective regional anesthesia technique characterized by ease of operation, high safety, and minimal impact on respiratory and circulatory functions (5–7). This retrospective study evaluates the effect of ultrasound-guided bilateral SAPB in selected pediatric patients undergoing pectus bar removal, specifically those identified as having higher pain sensitivity or risk of opioid-related complications based on clinical judgment, to determine its impact on postoperative analgesia and opioid use. While SAPB provides clear benefits, its use should be individualized rather than routinely applied to all patients.

## Materials and methods

This retrospective study was approved by the ethics committee of Beijing Children's Hospital ([2025]-E-034-R), in accordance with the Declaration of Helsinki. Given that the study involved only anonymized medical record data without additional interventions or data collection, the Ethics Committee waived the requirement for preoperative informed consent. This was a retrospective observational study, and no changes to clinical care were introduced based on research purposes.

Data were collected from the hospital's information system and anesthesia database on pediatric patients who underwent pectus bar removal under general anesthesia between September 2024 and February 2025. There were no restrictions on sex, and the included patients were aged 6–18 years with an ASA classification of I-II. Exclusion criteria included severe cardiopulmonary disease, psychiatric disorders, hepatic/renal dysfunction, major surgery other than the Nuss procedure, operative time of ≥2 h, or incomplete medical records. Patients were divided into two groups: the SAPB group (Group S, *n* = 30), where ultrasound-guided bilateral SAPB was performed based on clinical factors such as anticipated pain sensitivity, history of poor response to analgesics, or risk of opioid-related complications (e.g., history of respiratory issues or opioid sensitivity), and the control group (Group C, *n* = 46), who did not receive SAPB.

**Figure 1:**
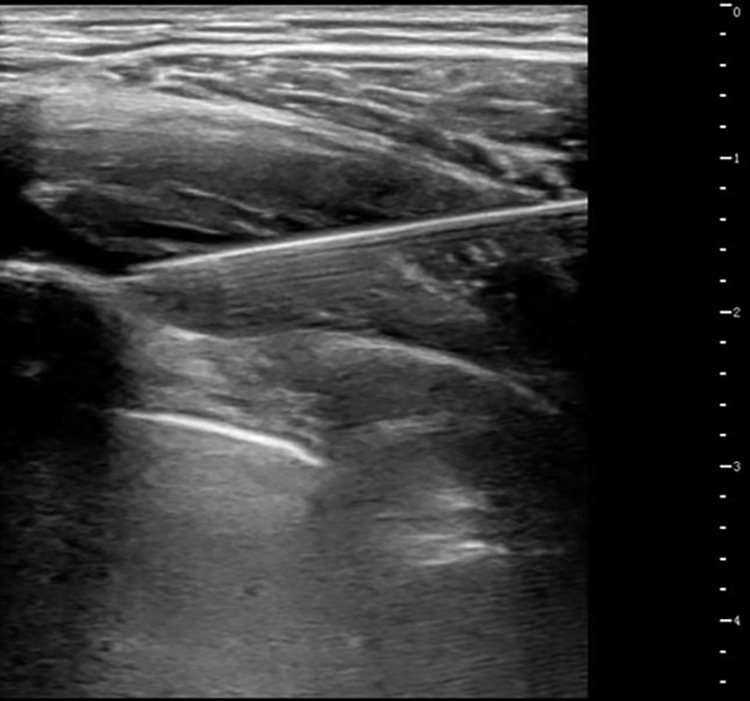
Ultrasound-guided SAPB. After injection, a hypoechoic fluid area is visible within the fascial plane between the inferior surface of the serratus anterior muscle and the superior surface formed by the ribs and external intercostal muscles.

Patients fasted for ≥6 h preoperatively without premedication. In the Group S, ultrasound-guided bilateral SAPB was performed in the pre-anesthesia room prior to entering the operating room. The procedure was as follows: the patient was placed in a lateral decubitus position, and a high-frequency linear ultrasound probe was used to locate the 5th rib at the midaxillary line. After skin disinfection, the probe was covered with a sterile sleeve and positioned horizontally. A puncture needle was inserted in-plane at a 30° angle to the skin, advancing from posterior to anterior. Under direct ultrasound visualization, the needle sequentially traversed the latissimus dorsi and serratus anterior muscles, reaching the fascial plane between the deep surface of the serratus anterior and the external intercostal muscle (see Figure 1). After confirming no blood aspiration, 2–3 ml of saline was injected to verify drug dispersion within the target fascial plane, followed by a slow injection of 0.3% ropivacaine at 0.5 ml/kg (maximum 20 ml per side). The patient then switched positions for contralateral blockade. All SAPB procedures were performed by two experienced anesthesiologists (each having completed ≥20 SAPB procedures), and surgery commenced approximately 15–20 minutes after block completion. In the Group C, no preoperative block was performed, and patients proceeded directly to the operating room.

Upon entering the operating room, all patients were monitored for SpO_2_, ECG, NIBP, and BIS. Anesthesia induction in the Group C consisted of intravenous propofol 2.5–3.5 mg/kg, sufentanil 0.3–0.5 μg/kg, and rocuronium 0.5 mg/kg after loss of consciousness. In the Group S, the sufentanil dose was reduced to 0.2–0.3 μg/kg, with other medications identical between groups. After muscle relaxation, a laryngeal mask airway was inserted, and mechanical ventilation was initiated using volume-controlled ventilation (respiratory rate: 14–20 breaths/min, tidal volume: 6–8 ml/kg, inspiratory-to-expiratory ratio: 1:2, FiO_2_: 50%), maintaining PETCO_2_ at 35–45 mmHg (1 mmHg = 0.133 kPa). Anesthesia was maintained with an intravenous propofol infusion 6–10 mg/kg/h to keep BIS values between 40 and 60. Remifentanil dosage was adjusted based on hemodynamic parameters (infusion rate: 0.1–0.4 μg/kg/min), maintaining blood pressure and heart rate fluctuations within 20% of baseline values. Fifteen minutes before the end of surgery, ibuprofen (10 mg/kg, maximum 400 mg) was administered intravenously. Five minutes before surgery completion, anesthetic infusions were discontinued. Sugammadex (2 mg/kg) was administered intravenously to reverse residual neuromuscular blockade postoperatively. Once spontaneous breathing was adequately restored, the laryngeal mask was removed, and patients were transferred to the post-anesthesia care unit (PACU) where they were observed for 30 min before returning to the ward.

## Data extraction

The following data were obtained from medical records: demographics **[**age, gender, body mass index (BMI), ASA, the number of pectus bars in the body], postoperative pain scores, anesthesia and surgical information (dosage of sufentanil during anesthesia induction, dosages of propofol and remifentanil during the maintenance phase of anesthesia, surgical duration, extubation time, total anesthesia duration) and postoperative complications.

Postoperative pain at rest and during coughing was assessed using the Numerical Rating Scale (NRS) at the following time points: immediately after awakening (Aldrete score ≥9), and at 1 h, 3 h, 6 h, 12 h, and 24 h postoperatively. If the resting NRS score immediately after awakening was ≥5, an additional dose of fentanyl (0.5–1 µg/kg) was administered for analgesia.

In Group S, the insertion time for serratus anterior plane block (SAPB) on each side (from needle-skin contact to complete needle withdrawal after injection) and number of puncture attempts (defined as withdrawing the needle from the skin or retracting it by >1 cm, counted as one attempt) were also noted.

Postoperative complications included incidence of hypoxemia (SpO_2_ < 90% under room air), nausea and vomiting, urinary retention, delayed oral intake (time to first postoperative oral intake>6 h), and delayed ambulation (time to first postoperative ambulation >6 h). In Group S, the puncture site was observed for adverse events such as infection, hematoma, or subcutaneous emphysema.

## Statistical analysis

Data were analyzed using SPSS 23.0 software (IBM Corp., NY, USA). The Shapiro–Wilk test was applied to assess data normality. Means and standard deviations (SD) were reported for normally distributed continuous data, and independent-sample *t*-tests were performed to compare group means. The Mann–Whitney U test was employed for non-normally distributed continuous data and presented as median (M) and interquartile range (IQR). Categorical data were analyzed using the *χ*² test or Fisher's exact test and expressed as counts (*n*) or percentages (%). For repeated measurements, such as NRS scores, *P* values from *t*-tests were adjusted using the Bonferroni correction to account for multiple comparisons (adjusted *α* = 0.008). A two-sided *P* < 0.05 was considered statistically significant for all other comparisons unless otherwise specified.

## Results

A total of 76 pediatric patients (30 in Group S and 46 in Group C) were included for data analysis. There were no statistically significant differences (*P* > 0.05) between the two groups in terms of age, gender ratio, BMI, surgical duration, anesthesia duration, and extubation time, as shown in Table 1. All pediatric patients in Group S successfully underwent ultrasound-guided SAPB (a total of 60 procedures), with a mean puncture time of 2.6 ± 1.8 min. In 15 of the blocks, two puncture attempts were required, while the remaining were successfully completed in a single attempt.

**Table 1:** Comparison of basic demographic characteristics between the two groups.

Variable	Group S (*n* = 30)	Group C (*n* = 46)	*P*
Age (years)	13.9 ± 2.6	13.3 ± 3.5	0.074
Gender			
Male	25 (83.3%)	39 (84.8%)	0.866
Female	5 (16.7%)	7 (15.2%)	
BMI (kg/m^2^)	18.2 ± 1.5	17.7 ± 2.6	0.457
Surgical duration (min)	36.2 ± 14.4	35.6 ± 21.7	0.905
Anesthesia duration (min)	60.0 ± 13.3	65.4 ± 21.7	0.296
Extubation time (min)	11.3 ± 5.5	12.7 ± 5.0	0.624

**Figure 2:**
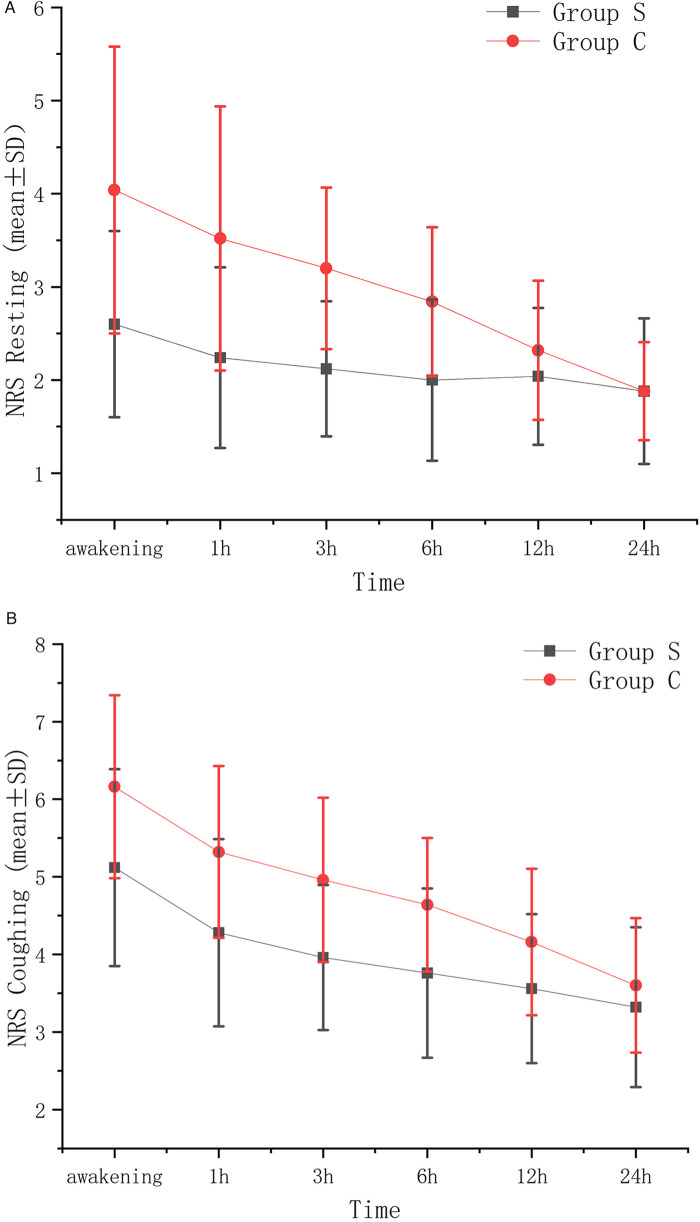
VAS resting **(A)** and VAS coughing **(B)** standard error graph between groups at different time points.

After Bonferroni correction (adjusted *α* = 0.008), the resting and coughing NRS scores within 6 h postoperatively in Group S were significantly lower than those in Group C (*P* < 0.008), as shown in Table 2. No significant differences were observed at 12 h or 24 h after correction, as shown in Table 2 (*P* > 0.008). Additionally, Figure 2 illustrated line graphs of NRS scores for resting and coughing states across six postoperative time points (immediately after awakening, 1, 3, 6, 12, and 24 h) in the Group S and the Group C, with standard error bars highlighting differences in pain control between groups.

**Table 2:** Comparison of VAS scores at various postoperative time points between the two groups.

Group	State	immediately after awakening	1 h postoperatively	3 h postoperatively	6 h postoperatively	12 h postoperatively	24 h postoperatively
Group S (*n* = 30)	Resting	2.6 ± 1.1	2.2 ± 1.0	2.1 ± 0.7	2.0 ± 0.9	2.0 ± 0.7	1.9 ± 0.8
Group C (*n* = 46)		4.0 ± 1.5	3.5 ± 1.4	3.2 ± 0.9	2.8 ± 0.8	2.3 ± 0.7	1.9 ± 0.5
*P*		0.000*	0.001*	0.000*	0.001*	0.188	1.000
Group S (*n* = 30)	Coughing	5.1 ± 1.3	4.3 ± 1.2	4.0 ± 0.9	3.8 ± 1.1	3.6 ± 1.0	3.3 ± 1.0
Group C (*n* = 46)		6.2 ± 1.2	5.3 ± 1.1	5.0 ± 1.1	4.6 ± 0.9	4.2 ± 0.9	3.6 ± 0.9
*P*		0.004*	0.003*	0.001*	0.003*	0.031	0.303

* *p* < 0.008, significant after Bonferroni correction for 6 comparisons (adjusted *α* = 0.05/6).

Compared to Group C, Group S exhibited reduced dosages of sufentanil and remifentanil, as well as a lower postoperative fentanyl usage rate (*P* < 0.05), as presented in Table 3. No significant differences were observed between the two groups in terms of the number of pectus bars or the dosage of propofol (*P* > 0.05), as shown in Table 3.

**Table 3:** Comparison of the number of pectus bars and usage of main anesthetic drugs between two groups.

Group	Pectus bars (1/2)	Sufentanil (μg/kg)	Propofol (mg/kg/h)	Remifentanil (μg/kg/min)	Fentanyl
Group S (*n* = 30)	19/11	0.26 ± 0.05	7.5 ± 1.2	0.18 ± 0.05	1 (3.3%)
Group C (*n* = 46)	35/11	0.41 ± 0.05	7.5 ± 1.0	0.22 ± 0.05	9 (19.6%)
*P*	0.231	0.000*	0.870	0.006*	0.041*

* *p <* 0.05.

All pediatric patients maintained stable intraoperative hemodynamics and were discharged within 24 h postoperatively. In Group S, no adverse events such as hematoma, infection, or subcutaneous emphysema at the puncture site were observed postoperatively. The incidence of postoperative hypoxemia in Group S was significantly lower than that in Group C (*P* < 0.05). There were no statistically significant differences between the two groups in terms of nausea and vomiting, delayed ambulation, or delayed oral intake (*P* > 0.05), as shown in Table 4.

**Table 4:** Comparison of postoperative adverse events between Two groups.

Group	Hypoxemia	Nausea and vomiting	Delayed ambulation	Delayed oral intake
Group S (*n* = 30)	4 (13.3%)	2 (6.7%)	2 (6.7%)	2 (6.7%)
Group C (*n* = 46)	16 (34.8%)	8 (17.4%)	8 (17.4%)	7 (15.2%)
*P*	0.038*	0.176	0.176	0.259

* *p <* 0.05.

## Discussion

This retrospective study demonstrates that ultrasound-guided bilateral SAPB significantly improved postoperative pain control in selected pediatric patients undergoing pectus bar removal, particularly those with anticipated higher pain sensitivity or risk of opioid-related complications. The SAPB group exhibited lower resting and coughing NRS scores within 6 h postoperatively, reduced intraoperative opioid use (sufentanil and remifentanil), and lower postoperative fentanyl supplementation compared to the control group. Additionally, the SAPB group had a significantly lower incidence of postoperative hypoxemia, likely due to decreased opioid-related respiratory depression. These findings suggest that SAPB is a valuable component of multimodal analgesia in carefully selected cases.

While pectus bar removal is generally associated with moderate pain, the intensity can vary based on patient-specific factors such as pain sensitivity, surgical trauma, or individual analgesic response. For patients with mild pain, systemic analgesics like ibuprofen may suffice, as noted by some reviewers (8, 9). However, in cases where patients are at higher risk of severe pain or opioid-related complications, SAPB offers a targeted approach to enhance analgesia while minimizing opioid use. The decision to perform SAPB in this study was based on clinical judgment, considering factors such as prior pain management challenges or risk of respiratory depression. This selective approach aligns with the principle of individualized pain management, as routine SAPB implementation may not be necessary for all patients undergoing pectus bar removal. While SAPB provides clear benefits in reducing postoperative pain and opioid use, we do not suggest routine implementation in all patients. Instead, its use should be individualized based on patient-specific factors such as pain sensitivity, previous analgesic response, and risk of opioid-related complications.

High thoracic epidural analgesia and paravertebral nerve blocks are widely used for postoperative pain management in thoracic surgery with proven effect (10). However, these techniques demand advanced technical skills and carry significant risks. Epidural analgesia may result in unintended dural puncture, epidural hematoma, or abscess formation, with substantial hemodynamic effects and potential respiratory depression (11). Paravertebral blocks, on the other hand, pose risks of pneumothorax and hemodynamic instability (12). Given that pectus bar removal causes relatively milder pain compared to other thoracic surgeries and is associated with rapid postoperative discharge, a simpler and safer regional block technique is preferable. Blanco et al. (5) first described the SAPB in 2013 as a method to achieve analgesia by blocking the lateral cutaneous branches of the intercostal nerves traversing the serratus anterior plane. The superficial target of this block avoids major blood vessels and nerves, making it a safe and straightforward procedure with minimal impact on thoracic sympathetic nerves and circulation. Multiple studies have confirmed its effect in providing postoperative analgesia for rib (6), video-assisted thoracoscopic (7, 13), breast (14, 15), and pectus excavatum repair surgeries (16). Our study similarly demonstrates that SAPB significantly reduces postoperative pain scores in children undergoing pectus bar removal procedure, decreases perioperative opioid consumption, reduces adverse effects such as postoperative nausea and vomiting (PONV), and promotes postoperative recovery. Regarding analgesic effect, studies have shown SAPB to outperform local anesthetic infiltration (17) and to be comparable to paravertebral blocks and erector spinae plane blocks (13, 18), establishing it as a recognized alternative for patients undergoing video-assisted thoracoscopic surgeries.

Clinically, SAPB can be performed as either a superficial SAPB (injecting local anesthetic into the fascial plane between the latissimus dorsi and serratus anterior) or a deep SAPB (injecting into the fascial plane between the serratus anterior and the external intercostal muscle/ribs). The comparative analgesic effect of these two planes remains debated. Blanco et al. (5) reported a broader dermatomal coverage with superficial SAPB, suggesting superior analgesia. However, Biswas et al. (19) injected methylene blue into both the superficial and deep planes of cadaveric specimens and found similar dye spread, hypothesizing comparable clinical effect. A meta-analysis by Singh et al. (20) of several clinical studies also concluded that the two approaches yield equivalent outcomes. Superficial SAPB can be technically challenging, as accurately positioning the needle in the soft fascial plane between the latissimus dorsi and serratus anterior is sometimes difficult, occasionally resulting in intramuscular injection (15). In contrast, deep SAPB allows rapid ultrasound-guided access to the rib surface beneath the serratus anterior; the needle is withdrawn slightly after contacting the periosteum, enabling faster injection with fewer attempts and reducing technical difficulty. Additionally, superficial SAPB may block the long thoracic nerve, potentially causing winged scapula (21), whereas deep SAPB avoids this nerve. Given its effective analgesia, simpler execution, and lower complication rate, deep SAPB was exclusively used in our hospital. The needle tip remained fully visible throughout all procedures to prevent accidental intercostal muscle puncture and pneumothorax.

As a fascial plane block, the volume of injected local anesthetic has a significant impact on the range of dermatomes blocked. Cadaveric studies have shown that a 40-ml volume results in far greater spread than 20 ml (19). Clinical research also indicates that 40 ml of local anesthetic in SAPB covers more dermatomes than 20 ml, though the duration of effective analgesia remains similar between groups (22). Most adult SAPB studies use local anesthetic volumes of 20–30 ml (20), but pediatric SAPB research is limited. Based on adult research experience and previous clinical trials, our hospital typically administers unilateral serratus anterior plane block with 0.3% ropivacaine at a dosage of 0.5 ml/kg (maximum dose: 20 ml), achieving satisfactory analgesia without evidence of local anesthetic toxicity or long thoracic nerve blockade, suggesting this volume is appropriate for pediatric patients. SAPB provides a broad block range, with a single T5 injection covering dermatomes T2-T9 (5). In this study, all chest wall incisions were located between the T4-5, T5-6, and T6-7 intercostal spaces, and SAPB at the T5 rib level allowed local anesthetic dispersion near the incisions, ensuring reliable postoperative analgesia. Compared to intercostal nerve blocks, a single SAPB effectively addresses multiple ipsilateral chest wall incisions (23), significantly reducing the number of required punctures.

This study has several limitations. First, its retrospective design may introduce selection bias and limit the ability to control for confounding variables, such as variations in surgical technique or postoperative care. Second, the sample size (*n* = 76) may have been insufficient to detect statistically significant differences in secondary outcomes, such as postoperative nausea and vomiting, delayed ambulation, and delayed oral intake. Third, pain assessments relied on the Numerical Rating Scale, which may be subject to variability in pediatric patients due to differences in pain perception and reporting. Finally, the study did not include long-term follow-up to evaluate the impact of SAPB on chronic pain or other delayed outcomes. Future prospective, randomized controlled trials with larger sample sizes and extended follow-up periods are needed to confirm these findings and further elucidate the benefits of SAPB in pediatric pectus bar removal surgery.

## Conclusion

Ultrasound-guided bilateral SAPB is a safe and effective component of multimodal analgesia for selected pediatric patients undergoing pectus bar removal, particularly those with higher pain sensitivity or risk of opioid-related complications. While SAPB provides clear benefits in reducing postoperative pain and opioid use, we do not suggest routine implementation in all patients. Instead, its use should be individualized based on patient-specific factors such as pain sensitivity, previous analgesic response, and risk of opioid-related complications. Further prospective studies are needed to optimize patient selection criteria and confirm these findings.

## Data Availability

The original contributions presented in the study are included in the article/Supplementary Material, further inquiries can be directed to the corresponding author.
